# A qualitative study to explore opinions of Saudi Arabian radiologists concerning AI-based applications and their impact on the future of the radiology

**DOI:** 10.1259/bjro.20210029

**Published:** 2022-03-21

**Authors:** Walaa Alsharif, Abdulaziz Qurashi, Fadi Toonsi, Ali Alanazi, Fahad Alhazmi, Osamah Abdulaal, Shrooq Aldahery, Khalid Alshamrani

**Affiliations:** ^1^ Department of Diagnostic Radiology Technology, College of Applied Medical Sciences, Taibah University, Madinah, Saudi Arabia; ^2^ Society of Artificial Intelligence in Healthcare, Riyadh, Saudi Arabia; ^3^ Department of Radiology, Faculty of Medicine, King Abdulaziz University, Jeddah, Saudi Arabia; ^4^ Radiography and Diagnostic Imaging, School of Medicine, University College Dublin, Dublin, Ireland; ^5^ Applied Radiologic Technology, College of Applied Medical Science, University of Jeddah, Jeddah, Saudi Arabia; ^6^ College of Applied Medical Sciences, King Saud bin Abdulaziz University for Health Sciences, Jeddah, Saudi Arabia; ^7^ King Abdullah International Medical Research Center, Jeddah, Saudi Arabia; ^8^ Ministry of the National Guard - Health Affairs, Jeddah, Saudi Arabia

## Abstract

**Objective:**

The aim of this study was to explore opinions and views towards radiology AI among Saudi Arabian radiologists including both consultants and trainees.

**Methods:**

A qualitative approach was adopted, with radiologists working in radiology departments in the Western region of Saudi Arabia invited to participate in this interview-based study. Semi-structured interviews (*n* = 30) were conducted with consultant radiologists and trainees. A qualitative data analysis framework was used based on Miles and Huberman’s philosophical underpinnings.

**Results:**

Several factors, such as lack of training and support, were attributed to the non-use of AI-based applications in clinical practice and the absence of radiologists’ involvement in AI development. Despite the expected benefits and positive impacts of AI on radiology, a reluctance to use AI-based applications might exist due to a lack of knowledge, fear of error and concerns about losing jobs and/or power. Medical students’ radiology education and training appeared to be influenced by the absence of a governing body and training programmes.

**Conclusion:**

The results of this study support the establishment of a governing body or national association to work in parallel with universities in monitoring training and integrating AI into the medical education curriculum and residency programmes.

**Advances in knowledge:**

An extensive debate about AI-based applications and their potential effects was noted, and considerable exceptions of transformative impact may occur when AI is fully integrated into clinical practice. Therefore, future education and training programmes on how to work with AI-based applications in clinical practice may be recommended.

## Introduction

The rapid advancement of artificial intelligence (AI) continues to gain interest in the healthcare sectors, particularly in radiology. There has been an extensive debate about AI-based applications and their potential effects on the future of radiology.^1^ The current advancement in AI-based applications such as machine-learning (ML) and deep learning (DL) algorithms has proven their effectiveness in administration tasks (*e.g.,* scheduling), diagnostic tasks such as objects detection (*e.g.,* detection of abnormalities) and image classifications (*e.g.,* classify tumours into benign or malignant) with efficient accuracy.^2,3^

There has been a considerable amount of fear raised among radiologists due to statements made by AI researchers where they have suggested that radiology as a career is in danger of extinction.^4,5^ The implication is that AI technology could eventually replace radiologists completely, and therefore they would be wasting their time training for a role that AI could perform more effectively than them in a few years’ time. It has been reported that AI will, at the very least, alter the working lives of radiologists. However, while certain tasks that currently exist in the radiology workflow will be performed more quickly and effectively by AI algorithms, one should not underestimate the complex nature of the radiologist’s role in general, which involves finding solutions to complicated issues.^6^ It would be pointless to resist AI and its undeniable advantages at this stage; rather, the focus should be on understanding the change and adapting it to improve the radiological workflow.^7^ Hricak (2016) and Pesapane et al. (2018) advised to work with researchers to make sure that AI is utilised in a sensible and safe manner. By doing this, radiologists will be able to ensure that AI improves processes in the field, while at the same time allowing the radiologists themselves to work more efficiently and effectively.^8,9^

Over time, AI will probably spread to other areas of radiology and indeed the work of other healthcare professionals.^10^ Hosny et al. (2018) claimed that it is essential that radiologists received appropriate AI software training, so that the results that are achieved are up to clinical standards. While it is true that vast quantities of medical images from PACS systems would be highly useful for AI training, this kind of “big data” requires what can be referred to as “curation” by humans, where various processes are carried out including stratification, segmentation and filtering to examine image quality, for example.^10^ If trainees rely on computers when interpreting the data, they might end up lacking necessary analytical skills to excel in their job. So, while AI may assist trainees and enhance their interpretations, their dependence on automated processes could result in serious errors at great cost to patients.^11^

An understanding of the implications associated with AI is crucial for medical practitioners, especially the meaning of the technology and its contribution to the radiology profession. To the best of the researchers’ knowledge, except for recent studies that have investigated Saudi radiologists’ knowledge about the role of AI-based applications in radiology,^12–16^ this is the first qualitative study to explore views of radiologists in Saudi Arabia regarding the future of AI in radiology.

## Methods

A qualitative approach was considered appropriate for this study as it allows the researchers to gain a cross-sectional overview of the radiologists’ opinions about the future of the radiology profession with AI-based applications, and to identify any factors that could potentially impact upon its application in Saudi Arabia. Saudi Arabian radiologists (consultants and trainees) were invited to participate in this study. Ethical approval (Reference Number: SREC/AMS 2020–62-DRD) was obtained from the relevant Institutional Review Board (IRB) in Saudi Arabia. Individual semi-structured interviews were conducted from July to September 2020 to investigate radiologists’ perceptions of AI-based applications and their impact on the future of radiology. This technique allowed radiologists to freely use their own words to express their feelings and provide in-depth information and detail regarding the topic.^17^ This study involved *n* = 30 individual semi-structured interviews with a purposive sampling of radiologists in the Western region of Saudi Arabia. The purposive sampling technique allowed the researchers to identify and select individuals with particular characteristics and experience relevant to the study.^18^ Although the current study involved the use of purposive sampling, a random approach was used in order to eliminate the risk of researchers’ bias in the selection of participants as recommended by Preece (1994).^19^ The interview questions were designed by the research team according to the literature and were guided by the topic guide (Table 1). They were validated through a pilot study, and minor revisions were made based on the pilot comments. The interview was conducted in English as all participants felt more comfortable speaking in English. The study participants were interviewed until data saturation was reached.

**Table 1.** Example of topic guide

Interview Questions Guide
1. Interviewee’s background:
Place of work, current position, area of expertise and experience
2. The use of Artificial Intelligence (AI) in medical imaging
Information about AI-based applications in radiology
Personal use of AI-based applications
Training on using AI-based applications
Involvement in AI development
Any factors or issues that would influence using AI, training, AI development
Resistance in using AI-based applications
3. Impact of AI on medical imaging in future
Future use of AI in medical imaging
AI implementation in the diagnostic imaging department
Radiologist’s career and experience
Performance
Specialisation with the AI
Likelihood of replacing radiologists’ job
Significant changes of AI on speciality
Potential AI mistakes
4. Education and training
Future medical education
Integrate AI practice in the education programme
Societies or governing bodies
Any factors or issues that would influence future medical students’ education
5. Any additional information

Individual permissions were obtained to record the interview responses using a digital recorder. All interview responses were recorded in order to ensure that the collected data were accurate, and their body language was observed, and to reflect the real phenomena being studied. Careful attention was paid by the researchers to the interviewees’ intonation and body language, as highlighted by Oppenheim (1998).^20^ Memos/manual maps were used during the stages of the analysis to provide a visible ‘audit trail’ as the analysis moved on from raw data and interpretation to writing up the findings.

**Figure 1.**
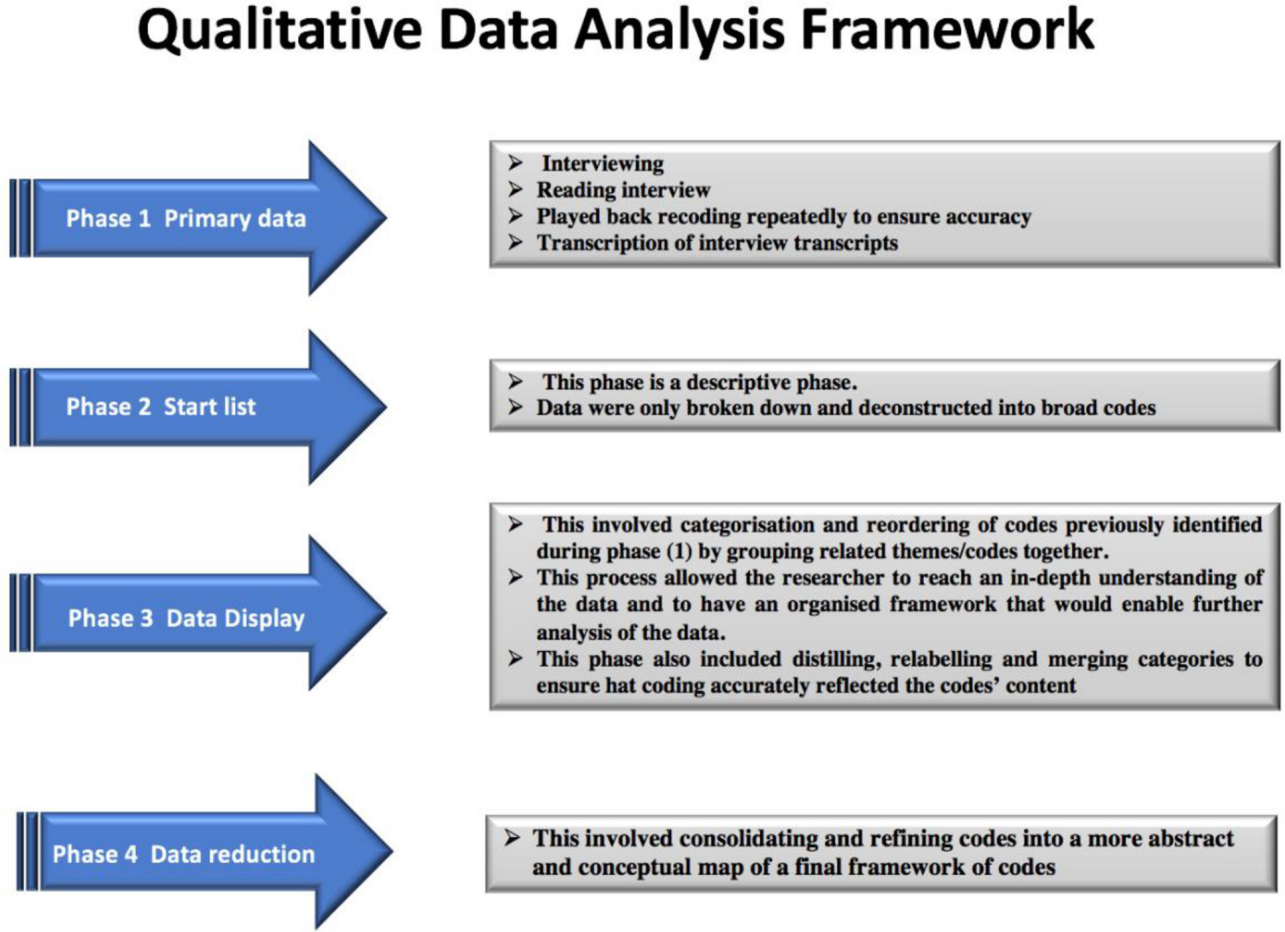
Qualitative framework: Miles and Huberman to ensure that the interview is efficient and that the data gathered are as rich, accurate and close as possible to reflecting the real phenomena being studied.

To minimise interview bias, and as recommended by Polit and Beck (2004), participants were not pressured to respond quickly to the questions during the interview.^21^ Equal time opportunity was given for questions to make sure that all participants’ voices were heard. The recorded interviews were manually transcribed by the researcher. The participants were given the opportunity to review their transcriptions so they could clarify points if they wanted to, and to ensure that there was no misinterpretation to what they said. No edits or changes were made by the participates to the transcripts. The study participants’ identities were not specified within the transcript document, as each participant was assigned a code known only to the researchers. Therefore, participants in this study were not identifiable, except to the researchers who had interviewed them. The results were subjected to qualitative analysis using designed qualitative software (*Nvivo 12*). Responses were coded, and common themes were extracted, as demonstrated in Figure 1, using a data-led approach that is based on Miles and Huberman’s framework.^22^

## Results

**Figure 2.**
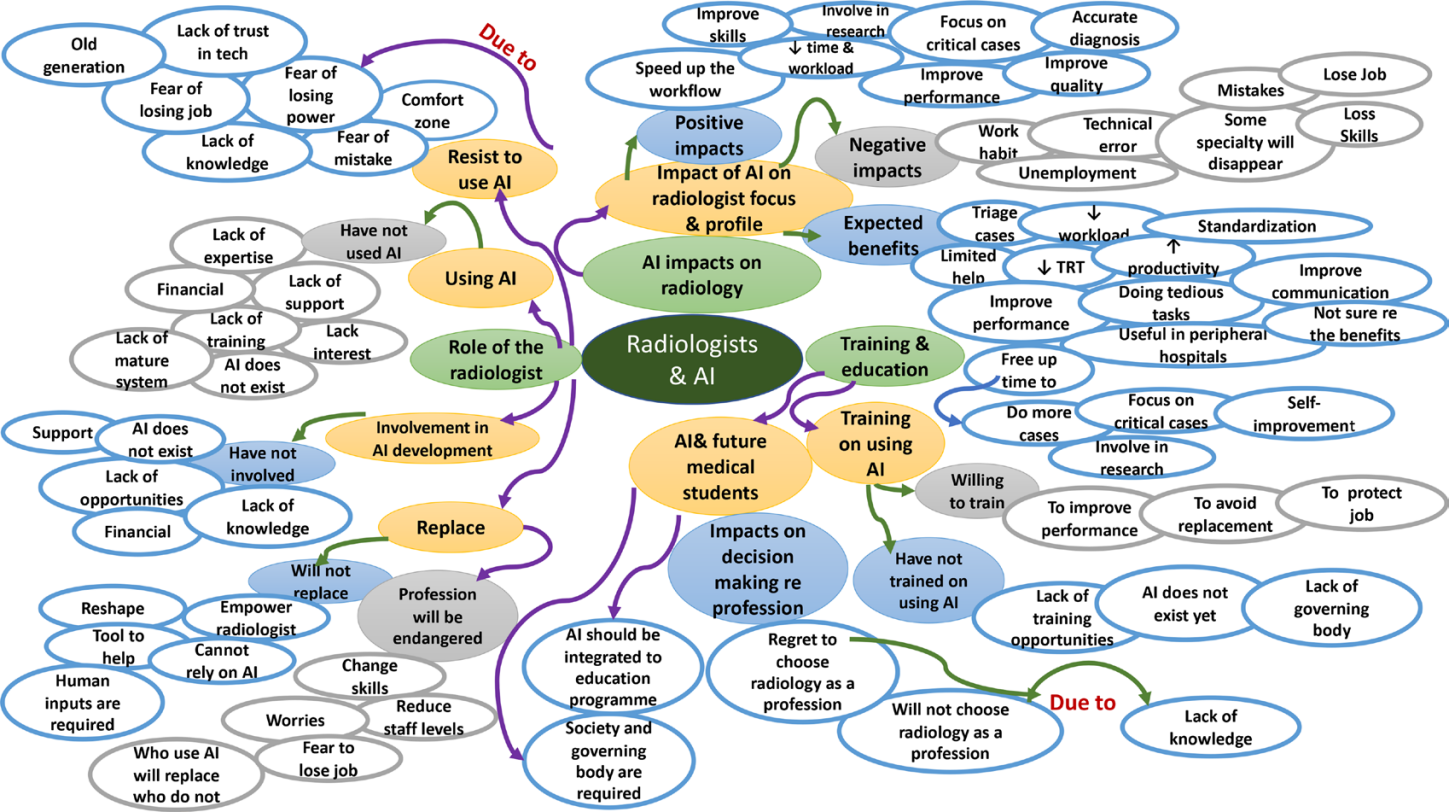
Conceptual map for the future of AI-based applications and their impact on the radiology profession

In total, 30 radiologists were recruited and participated in this study, distributed as follows: consultants *n* = 15 and trainees *n* = 15. Thirteen (43%) of the participants were female and 17 (56%) were male. The participants’ experience varied; 18 (60%) of the study participants had five years or less and 12 (40 %) of the participants had more than five years of experience. The participants who were interviewed were from radiology departments in the Western region of Saudi Arabi. The identified themes included^1^: the role of radiologists,^2^ the impact of AI on radiology, and^3^ education and training. These three themes comprised of several subthemes that emerged from the interview responses (Table 2). These themes and subthemes were reviewed and mapped to develop a conceptual map (Figure 2). The conceptual map indicated that several factors, such as lack of training and support, were attributed to the non-use of AI-based applications in clinical practice and the absence of radiologists’ involvement in AI development. Despite the expected benefits and positive impacts of AI on radiology, a reluctance to use AI-based applications might exist due to a lack of knowledge, fear of error and concerns about losing jobs and/or power. Medical students’ radiology education and training appeared to be influenced by the absence of a governing body and training programmes. This will be considered further in the discussion section.

**Table 2.** The main themes and sub-themes

Theme and Sub themes	Participants Coded	Citation Coded
Theme1-Role of Radiologists	30	1156
Sub theme: Using AI-based applications in clinical practice of radiology	30	300
Sub theme: Responsibility in instances of potential AI-based applications mistakes	30	286
Sub theme: AI-based applications replacing radiologists	30	302
Sub theme: Radiologist involvement in AI development	30	270
Theme2- AI impact on radiology profession	30	700
Sub theme: Significant changes of AI-based applications on radiology	30	139
Sub theme: Impact of AI-based applications on radiologist focus & profile	30	103
Sub theme: Benefits of the AI-based applications	30	458
Theme3- Education and Training	30	320
Sub theme: Training on using AI	30	140
Sub theme: AI & future medical students	30	180
**Total**	**30**	**2176**

## Discussion

There has been extensive discussion recently concerning AI-based applications and their potential impacts on the medical field and, in particular, on radiology.^23–27^ A total of 2,176 comments were provided by the study participants in relation to AI-based applications and their impact on the radiology field in Saudi Arabia. All study participants reported that they have not used AI-based applications in their clinical practice, which is in accordance with Tajaldeen et al.(2020).^13^ . However, reasons behind not using AI were not indicated by the researchers.^13^ In this study, several factors were identified as barriers to radiologists from using AI, including financial issues, lack of regulations, lack of support, lack of training, lack of AI-based applications and expertise. These factors could delay the potential adoption of AI in radiology.^28^ All participants also reported a lack of training in using AI-based applications, and the majority showed a willingness to participate in training sessions:

*“We need to follow the technology and advancement in our field. I am very motivated and interested in spending time learning how to use AI in order to improve performance and protect careers in the future.”* (Participant [16]: Radiologist trainee)

Despite this great sense of motivation among radiologists to train on using AI, a few trainees reported that they need more time to prepare for training in the use of such intelligent technology. This could indicate their readiness to enhance their work by using AI-based applications when they have a solid background in their profession:

*“As a young radiologist, it is still too early to think about AI applications and how to use it, I need to spend more time in reporting and discussing cases to shape the future of my profession.”* (Participant [23]: Radiologist trainee)

In addition, some participants were unconvinced about the potential use of AI-based applications in radiology, as it is a long way from being ready for daily use in radiology:

“*As far as I can see, the implementation of AI is only focused on image screening such as chest X-ray. Until now, I cannot be convinced or believe that AI will be used in daily work in radiology. Reporting and reading process are a challenge, and need back and forth discussion and communication with physicians.”* (Participant [11]: Consultant radiologist)

This concern might lead to questions about whether radiologists are unsure yet regarding the potential use of AI-based applications in radiology or uncomfortable about their future and the impact of AI on their career. Experts in the field still agree that the complex work of radiologists cannot be managed by AI-based applications alone.^1,29–31^ However, a bigger potential threat from AI-based applications on the career of radiologists was noted.^32,33^ A trainee in the current study revealed significant anxiety concerning the uncertain impact of AI-based applications on their radiology career:

*“Umm... honestly, I am a bit worried, and not sure if AI would be able to do the same work as human, or if it will replace radiologists in the future.”* (Participant [19]: Radiologist trainee)

Other trainees also revealed the same worries regarding job replacement. The interview findings showed the experiences that the trainees made during their studies where some lecturers and/or doctors play a role in discouraging medical students from considering radiology as their future profession:

*“Some doctors advised me to think twice about my job career decision (radiology), as the future of AI in radiology is not clear yet, it might replace most of the radiologists’ work, and the profession might be endangered.”* (Participants [30]: Radiologist trainee)

Contrary to losing jobs concerns that the trainees revealed in this study, it was reported that radiologists and medical students believe that AI-based applications will not replace radiologists.^13,34,35^ This is similarly determined by the consultant radiologists in the current study, who agreed that AI-based applications will transform the current practice in radiology (*e.g*., take over the routine and tedious cases), yet it will not replace radiologists as human input is inevitable. This is supported by Eltorai et al. (2017) who reported that a number of tasks can be automated or improved with AI-based applications.^36^ In contrast, Hoek et al. (2020) claimed that certain specialities in radiology might disappear and be replaced by AI-based applications in the future.^32^ A recent study showed that radiologists would choose an interventional radiology subspeciality more than other subspecialities.^37^ It seems that radiologists believe that AI will not substitute those who work in interventional departments. Some participants in this study showed regret in rushing their career choice. This situation is in contrast to that in a recent study, where literature evidence showed that most of the participants had no plan to change their profession and/or speciality due to the AI^13^ :

*“I regret choosing radiology as a profession, as I think radiology will be in danger in 10 years (such as general radiologist) when we start to rely on the AI.”* (Participant [23]: Consultant radiologist)

Despite a convincing discussion in the published literature regarding the beneficial effects of AI-based applications on radiologists’ daily work,^38,39^ some participants reported that resistance to AI-based applications might be intensified by their perceptions of losing their jobs, losing power, feeling out of their comfort zone, misconceptions and fear of making mistakes. In contrast, other participants believe that *‘It is not wise to resist tools that can help you in work and increase efficiency’*. It is expected that radiologists will show resistance against change in their clinical practice, as they might be alarmed by the introduction of such new technology such as AI, which could be the reason behind their anxiety about their future. Therefore, a proper training programme that focuses on learning how to adapt an AI-based application in clinical practice of radiology might contribute to reduced potential anxiety and resistance:

*“Well, usually we have those people who resist change and technology. The reason for this resistance to technology may be their perception of losing power, identity, and fear to change in work habit.”* (Participant [17]: Radiologist trainee)

The current study showed that the concerns and uncertainties associated with the future of the radiology profession is attributed to a lack of knowledge and information about the future radiology and AI. All the information they had gained about AI was acquired from stream media rather than formal education. This is in line with Ooi et al.(2021).^37^ who referred to the lack of understanding of the AI concept among residents and faculty radiologists.^37^ However, other researchers found that some participants had already heard about AI from scientific articles and/or university lectures.^13,34^ Several participants reported that this advancement in radiology may attract those who are more interested and open to computer science activities and technology (*e.g.,* programming, software developing), which is in line with Gong et al. (2019).^35^ , Hoek et al(2019).^32^ and Ooi et al (2021).^37^.^32,35,37^

The study participants insisted on the necessity of integrating the principle of AI into education programmes to educate them about the benefits and potential consequences of AI. This is supported by several studies that highlighted the need to integrate AI early in the medical educational programme, particularly in residency programmes, which could explain the uncertainty around using AI in clinical practice.^13,32,37,40^ Sit et al.(2020).^41^ referred to the essential knowledge that is required to understand AI-based applications, which includes the principles of AI, nomenclature and limitations.^41^ Students who gain more knowledge about AI worry less about the impact of AI on radiology.^41,42^ This suggests that organisations and governing bodies need to plan for the education and skills required to use AI effectively and safely. Understanding this topic would be beneficial for radiologists’ future careers.^41^

In terms of the involvement in AI development and research, a recent study by Ooi et al.(2021)^37^ found that only 17% of radiologists are involved in AI research while 67% showed an interest in getting involved in AI research.^37^ The interview findings in the current study indicated that the majority of participants have not been involved in the development of AI-based applications. Only one radiologist reported involvement in an AI-based application development project. Such involvement could help improve radiologists’ understanding of AI:

*“I am working with computer scientists’ team in developing AI system. As a radiologist I help them with providing data (images), review the cases, labelling and validation.”* (Participant [8]: Consultant radiologist)

In comparison between consultant radiologists and trainees’ responses in relation to the role of radiologists in AI-based application development, the former believe that consultant radiologists must play a role in AI-based application development. On the other hand, trainees think this task is beyond the scope of radiologist role. This is contrary to the European Society of Radiology survey (ESR), in which all of the radiologists agreed on the importance of radiologists’ role in AI development, especially during AI training and data validation.^43^ This may stem from the cultural issue among Saudi radiologist trainees who showed during the interview process that they would prefer to focus on their clinical skills more than physics and technical skills.

A study conducted by Tajadeen et al. (2020) found that few participants believe that AI will not change radiology field.^13^ In contrast, the findings derived from the current study revealed that radiologists foresee that AI could have positive and negative changes/impacts on their daily duty and future focus. Typical responses included, *“The needs will change”*, *“Skills and qualification requirements to hire radiologists will change”*, *“The way of working will change”*. The participants reported that AI-based applications might help radiologists to improve the delivery of care to patients, as AI-based applications can take over tedious and repetitive tasks (*e.g*., counting and measuring lesions) that can hamper productivity. Similar findings were shown by the ESR(2019)^11^ survey, in which the participants demonstrated positive perceptions of AI-based applications in relation to saving time and improving communication.^43^ In addition, most of the participants believe that AI-based applications will offer them an opportunity to expose more to the cases outside their subspecialities ; this is in line with the ESR report (2019)^43^ :

*“I think AI might help radiologists to interpret studies outside of their specialisation, if they want to work somewhere else part time to make more money. However, AI should help radiologists to improve their performance by providing the opportunity of learning new skills and focusing more on critical and complex cases”*. (Participant [8]: Radiologist trainee)

Conversely, conflicting views have been expressed by interviewees in this study, radiologists reported that AI-based applications may prompt unemployment, medical and/or technical errors, and may lead to careless work habits. This is consistent with Rubin(2019)^44^ who claimed that relying on AI-based applications could minimise radiologists’ attention and conscious competence.^44^ The potential increases in workload and costs due to maintenance and data analysis associated with AI-based applications implementation was also reported in the literature.^35,41,45^ However, the ESR (2019) study showed that radiologists were uncertain about the impact of AI on reporting workloads and job opportunities.^43^ This doubt about the impact of AI on radiology professionals may reflect the limited use of AI-based applications in clinical radiology practice.

The literature demonstrated that AI tools might cause potential mistakes, such as medical errors, inherent biases implication and ethical issues.^11,23,46^ The argument concerning responsibility in cases of AI mistakes is controversial.^9,47^ The ESR study (2019) agreed that the responsibility of AI usage is sharable between designers and users.^43^ In the current study, some participants were reluctant to assume responsibility in the instances when potential mistakes occurred due to the use of AI. The trainees in this study reported that AI developers (*e.g*., companies) should take responsibility in case of potential AI mistakes, and these findings are in line with Jungmann (2020).^33^ However, consultant radiologists in this study believe that radiologists should be liable for potential AI mistakes that might affect radiological diagnosis and patient treatment, as they cannot fully trust AI-based applications at the patients’ own risk. This is consistent with Jungmann et al. (2020).^33^ The lack of understanding of the process behind the AI-based applications could apparently cause difficulty in taking responsibility in case of error.^48^

This uncertainty among participants raises a critical question concerning accountability and liability in cases of misdiagnosis and/or improper treatment recommendations. Another question may be raised regarding patients’ willingness to accept reports issued by AI-based applications without the approval of a radiologist, as radiologists need to learn how to use the AI-based applications for healthcare delivery, and patients should be ensured that they will not be harmed by AI-based application. This warrants further investigation regarding the perceived benefits and risks of AI-based applications among consumers (*e.g*., patients). Ethical use of AI-based applications in radiology will require stakeholders to carefully consider how the AI approach is developed, and the way these tools are used and validated according to clear laws and regulations.^48,49^

Responses indicated that regulations and ethical frameworks need to be in place to help steer technological development in radiology and to determine the rules and responsibilities for the use of AI-based applications in an effective and safe manner. To the best of the researchers’ knowledge, no evidence related to the liability or accountability of AI system in radiology exists, and this lack of accountability raises the potential safety issue of using unverified or unvalidated tools in the clinical practice.^50^ Challenges in introducing AI-based applications in radiology would need to be regulated across different countries.^43,48^

## Limitations

The potential bias in any qualitative research study may be questionable. Bias in qualitative research can occur due to the way the question is proposed, the approach with which the participants respond and how the researchers prejudge. Being Saudi radiology professionals helped the researchers to understand the participants and their feelings towards AI and its future impact on radiology in Saudi Arabia more effectively than other researchers would be able to. This can be seen as a positive bias that the researchers brought to the study. Additional limitations of this study relate to the fact that it only involved the Western region of Saudi Arabia; however, the policies and procedures are similar in the radiology departments across the country. An additional limitation is that not all participants in qualitative research may say truthfully what they actually believe or do in practice. One of the reasons for providing incorrect information is social desirability bias. However, the researchers assumed that the research participants provided credible information, because they are professionals and have a vested interest in the topic of this study.

## Conclusion

This study aimed to explore the opinions and views of Saudi Arabian radiologists (consultants and trainees) on AI applications and their impact on the future of the radiology profession. The findings demonstrated that participants who do not use AI-based applications are willing to learn to be trained on the use of AI-based applications in their clinical practice. Several factors were attributed to the non-use of AI-based applications, such as financial issues, lack of regulations, lack of support, as well as the lack of AI-based applications and expertise. Radiologists reported that AI could have advantages and limitations on their daily work; they believe that it could change the current work in radiology departments, but it will not replace them. Despite the expected benefits of AI-based application implementation in radiology, the results indicated that radiologists might resist AI technology, and future medical students may avoid the radiology profession. The results of this study support the establishment of a governing body or national association to work in parallel with universities in monitoring training and integrating AI into the medical education curriculum and residency programmes.
